# The Use of Fiber-Reinforced Scaffolds Cocultured with Schwann Cells and Vascular Endothelial Cells to Repair Rabbit Sciatic Nerve Defect with Vascularization

**DOI:** 10.1155/2013/362918

**Published:** 2013-12-30

**Authors:** Hongyang Gao, Yang You, Guoping Zhang, Feng Zhao, Ziyi Sha, Yong Shen

**Affiliations:** ^1^Department of Orthopedics, The First Hospital of Hebei Medical University, Shijiazhuang 050031, China; ^2^Department of Cardiovasology III, The First Hospital of Hebei Medical University, Shijiazhuang 050031, China; ^3^Department of Spinal Surgery, The Third Affiliated Hospital of Hebei Medical University, Shijiazhuang 050051, China

## Abstract

To explore the feasibility of biodegradable fiber-reinforced 3D scaffolds with satisfactory mechanical properties for the repair of long-distance sciatic nerve defect in rabbits and effects of vascularized graft in early stage on the recovery of neurological function, Schwann cells and vascular endothelial cells were cocultured in the fiber-reinforced 3D scaffolds. Experiment group which used prevascularized nerve complex for the repair of sciatic nerve defect and control group which only cultured with Schwann cells were set. The animals in both groups underwent electromyography to show the status of the neurological function recovery at 4, 8, and 16 weeks after the surgery. Sciatic nerve regeneration and myelination were observed under the light microscope and electron microscope. Myelin sheath thickness, axonal diameter, and number of myelinated nerve fiber were quantitatively analyzed using image analysis system. The recovery of foot ulcer, the velocity of nerve conduction, the number of regenerating nerve fiber, and the recovery of ultrastructure were increased in the experimental group than those in the control group. Prevascularized tissue engineered fiber-reinforced 3D scaffolds for the repair of sciatic nerve defects in rabbits can effectively promote the recovery of neurological function.

## 1. Introduction 

Peripheral nerve injuries are common in clinical practice [1]. Peripheral nerve injuries can arise from trauma, cancer, or congenital defects [2, 3]. The regeneration of the injured nerve is slow and can result in complicated rehabilitation. Peripheral nerve injuries are challenging clinical issues to address. While short gaps less than 10 mm can be reconnected surgically with microsutures [4] or various nerve guidance channels [5–7], longer defects are more difficult to treat [8]. Autologous nerve graft is widely used in clinical surgery for nerve reconstructions and has been considered as the golden standard [9–11]. However, it still has several defectives including donor tissue availability limitation, extra incision, sacrifice of the donor nerve and danger of neuroma forming, multiple surgical sites, and possible size mismatch. Other options include allografts and xenografts, but those run the risk of disease transmission and immune rejection. Thus, there is a significant clinical need to address critical size nerve defects.

Nerve tissue engineering is a promising approach that has shown potential to address this need with synthetic nerve conduits. There has been a significant effort dedicated to developing synthetic nerve conduits that have resulted in encouraging regeneration and some degree of functional recovery of peripheral nerve defects without sacrificing other nerves [12]. However, simply bridging the transected nerve with an empty conduit would not satisfy the functional recovery. Numerous experiments indicated that, after nerve injury, oriented regrowth guidance and regeneration promoting factors played very important roles in nerve regeneration and functional recovery [13–15]. Schwann cells are closely associated with, and play key roles in, the development, maintenance, and regeneration of peripheral neurons [16–19]. The microenvironment of the conduit mimicking the biological situation is a vital issue in tissue regeneration. *In vivo*, nearly all tissues are supplied with nutrients and oxygen by a highly branched system of larger blood vessels, which are subdivided in the tissue into small capillaries, including the nerve system. The strategy based on the endothelial cells and their ability to form new vessels known as neoangiogenesis had been developed for the formation of the vessel system *in vivo*. Biomaterials with structural similarity to native extracellular matrix (ECM) have been shown to improve cell function.

In this paper, a new-typed fiber-reinforced 3D scaffold which had been demonstrated good biocompatibility *in vivo* [20, 21] was prepared to mimic ECM due to the critical role of the ECM in regulating cell function, acting as a nerve conduit, providing a permissive chamber for nerve regeneration in the rabbit sciatic nerve model. The 3D scaffold was cocultured with Schwann cells and vascular endothelial cells in order to support the vascularization and induce the nerve regeneration of the rabbit sciatic nerve. Two groups (nonvascularized and vascularized nerve tissue engineering scaffolds) were used to evaluate results of nerve regeneration.

## 2. Materials and Methods 

### 2.1. Materials

M199 medium (Gibco), DMEM medium (Gibco), trypsin (Gibco), nerve growth factor (Xiamen Bioway Biotech Co., Ltd., China), fetal bovine serum (Hangzhou Sijiqing Biological Engineering Materials Co., Ltd., China), rabbit anti-S-100 protein antibody, TritonX-100 (Sigma, USA), vascular endothelial growth factor (Xiamen Bioway Biotech Co., Ltd.), and Chitosan fibres (80% deacetylated; diameter, 12.5 *μ*m; tensile strength, 550 MPa) were obtained from Donghua University, China. PLLA was purchased from the Shandong Medical Appliance Factory, China, while Type I collagen (at a concentration of 1%), dioxane, and other chemicals were purchased from the Beijing Chemical Company Ltd, China. A total of 36 healthy adult New Zealand rabbits of both genders and one New Zealand rabbit (pregnancy on day 28) were provided by the Experimental Animal Center, Hebei Medical University, China.

### 2.2. Methods

#### 2.2.1. Preparation of the Biodegradable Fiber-Reinforced 3D Scaffolds

The biodegradable fiber-reinforced 3D scaffolds were prepared in accordance with a previously published method by Li et al. [20]. The composites were cut into the appropriate size matching the nerve defect and sterilized by Co60.

#### 2.2.2. Preparation of Nonvascularized and Vascularized Tissue Engineered Scaffolds

Sciatic nerve of fetal rabbits at 28 days of pregnancy was obtained. Schwann cells were cultured using two enzymatic digestions. Schwann cells of passage 2 at a concentration of 1 × 10^8^/L were separately injected into both ends of the scaffolds with a 100 ul microsyringe. Thereby, nonvascularized tissue engineered scaffolds were obtained.

Thoracic aorta of fetal rabbit was obtained. Vascular endothelial cells were cultured using enzymatic digestion. Schwann cells and vascular endothelial cells of passage 2 at a ratio of 2 : 1 were cultured in DMEM containing 20% fetal bovine serum and M199 medium. By the same method, cell suspension was infused into the scaffolds (Schwann cell at a concentration of 1 × 10^8^/L), forming vascularized tissue engineered scaffolds.

#### 2.2.3. Establishment of Animal Models

Experimental animals were randomly assigned to control group (nonvascularized tissue engineered scaffolds) (*n* = 18) and experimental group (vascularized tissue engineered scaffolds) (*n* = 18).

All operations were done under aseptic conditions. Abdominal anesthesia and nerve suture were done by the same person. A 20 mm segment of sciatic nerve was transversely excised at about 5 mm from proximal end of knee joint of left hind limb of rabbits. Under the operating microscope, the fiber-reinforced scaffolds cocultured with vascular endothelial cells and Schwann cells were used for defect repair with nontraumatic suture as the experimental group while the fiber-reinforced scaffolds cultured with only Schwann cells were utilized as control.

### 2.3. Detection Methods and Indices

#### 2.3.1. General Observation

Conditions of all over the body and wound were observed at various time points after surgery in both groups to compare foot ulceration and healing. During sampling, we paid great attention to whether there was a neuroma at the anastomotic site and to the adhesion condition between nerve graft and surrounding tissue.

#### 2.3.2. Determination of Nerve Conduction Velocity

At 4, 8, and 16 weeks after nerve transplantation, sciatic nerve was exposed in the second operation in both groups. Stimulating electrodes were inserted on the proximal and distal ends of nerve graft. Recording electrodes were inserted in the tibialis anterior muscle belly. The same stimulus intensity and frequency were given. The latency and conduction velocity were measured with MEM-3210 electromyography.

#### 2.3.3. Measurement of Wet Weight of Tibialis Anterior Muscle

At 4, 8, and 16 weeks after nerve transplantation, tibialis anterior muscle at operation side was incised, and blood on its surface was absorbed with blotting paper. Wet weight of tibialis anterior muscle was weighed with an electronic balance. Paired comparison was done.

#### 2.3.4. Observation with the Light Microscope

At 4, 8, and 16 weeks after nerve transplantation, a 4 mm middle segment of nerve was obtained from both groups and equally divided into two parts (distal, proximal, each 2 mm). Proximal segment of the nerve was fixed in 10% formaldehyde, embedded in paraffin, and sliced into transverse sections. These sections were subjected to Masson staining. Morphology of myelin sheath, axonal number and distribution, and connective tissue structure was observed under the light microscope. Images were analyzed using high-definition color image processing system (Institute of Beijing University of Aeronautics and Astronautics, China). Observation index was the number of nerve axon.

#### 2.3.5. Observation with the Transmission Electron Microscope

Distal segment of the nerve obtained at various time points was fixed in 4% glutaral, dehydrated with gradient alcohol and acetone, embedded with epoxy resin, and then sliced with an ultramicrotome. These sections were stained with uranyl acetate and lead citrate. Transmission electron microscope (H/7500, Hitachi, Japan) was utilized to observe axon diameter, myelin sheath distribution, lamellar structure, microtubule, mitochondrion, and microfilament. Observational indexes were axon diameter and myelin sheath thickness.

#### 2.3.6. Statistical Analysis

Data were analyzed with SAS software and expressed as mean ± SD. The difference between the two groups was compared with *t*-test. A value of *P* < 0.05 was considered statistically significant.

## 3. Results

### 3.1. General Observation of Animals

No apparent rejection was observed all over the body or in the operative field after allograft nerve complex transplantation in both groups. No noticeable infection appeared at the operative incision. At 2 weeks after operation, ulcer at different degrees was visible on the foot of the operation side. However, compared with the control group, at 5-6 weeks after operation, ulcer healing was better at the operation side in the experimental group. At 15-16 weeks after surgery, hindlimb braced in both groups and normal gait appeared in some animals.

Specimen collection during the secondary operation: tissue engineered scaffolds connected well to the host in both groups. No obvious neuroma was observed at the anastomotic site. There was no severe adhesion between the tissue engineered scaffolds and surrounding tissue and vascular network formed in the outer membrane.

### 3.2. Detection Results of Nerve Conduction Velocity

At 4 weeks after nerve transplantation, the animals of both groups did not respond to electrostimulation, with the absence of nerve conduction. Electromyogram showed a straight line. At 8 and 16 weeks after operation, nerve conduction velocity was restored to different degrees under the same stimulus intensity and frequency in both groups. Results revealed that the recovery of nerve conduction velocity was better in the experimental group than that in the control group (*P* < 0.05; Table 1).

### 3.3. Measurement of Wet Weight of Tibialis Anterior Muscle

The weight of animals in both groups was similar at various time points after nerve transplantation. Tibialis anterior muscle atrophy was obvious at the operation side in both groups at 4 weeks after operation. However, no significant difference in the wet weight of tibialis anterior muscle was detected between the two groups at 4 weeks (*P* > 0.05). The wet weight of tibialis anterior muscle increased at 8 and 16 weeks compared with that at 4 weeks in both groups to different degrees, indicating that the recovery was better in the experimental group than that in the control group (*P* < 0.05; Table 2).

### 3.4. Observational Results under the Light Microscope

**Figure 1:**
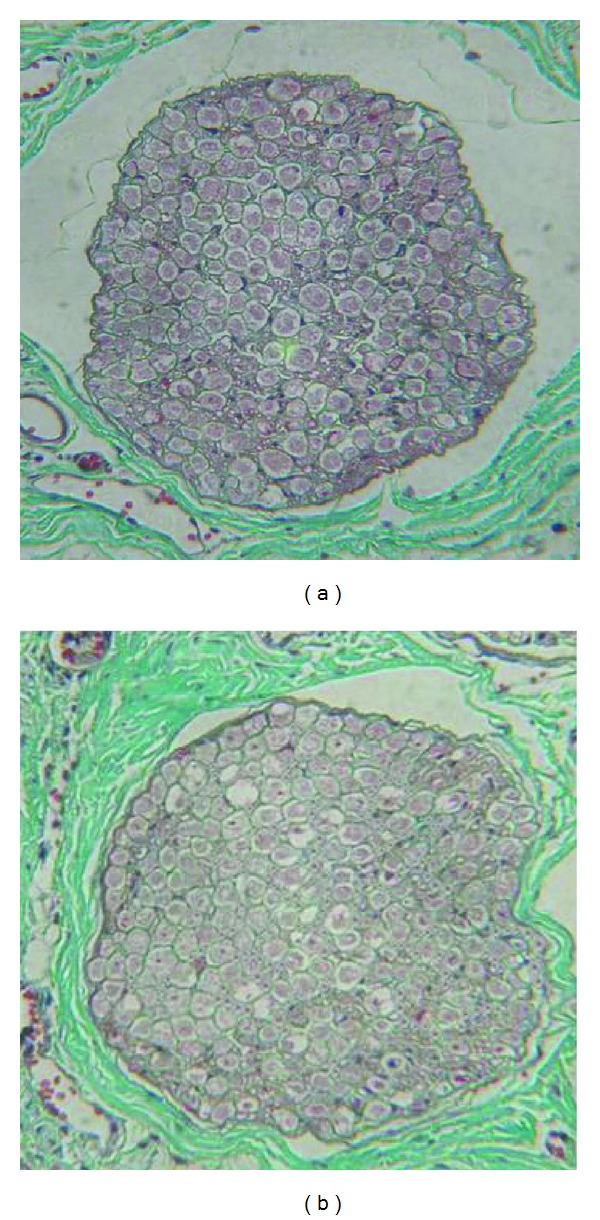
The image of sections under light microscope after Masson staining at week 16 (Masson ×400) ((a) control group, (b) the experimental group).

The sections were observed under light microscope after Masson staining. The section dyed at week 16 can be seen in Figure 1. Masson staining revealed that the number of regenerating nerve per unit area was higher in the experimental group than that in the control group at 4, 8, and 16 weeks after operation. Compared with the control group, regenerating nerve arranged regularly uniformly, with a few connective tissues and a high proportion of thick fibers. There were significant differences in the number of regenerating nerve axons per unit area in both groups at 4, 8, and 16 weeks (*P* < 0.05; Table 3).

### 3.5. Observational Results under the Transmission Electron Microscope

**Figure 2:**
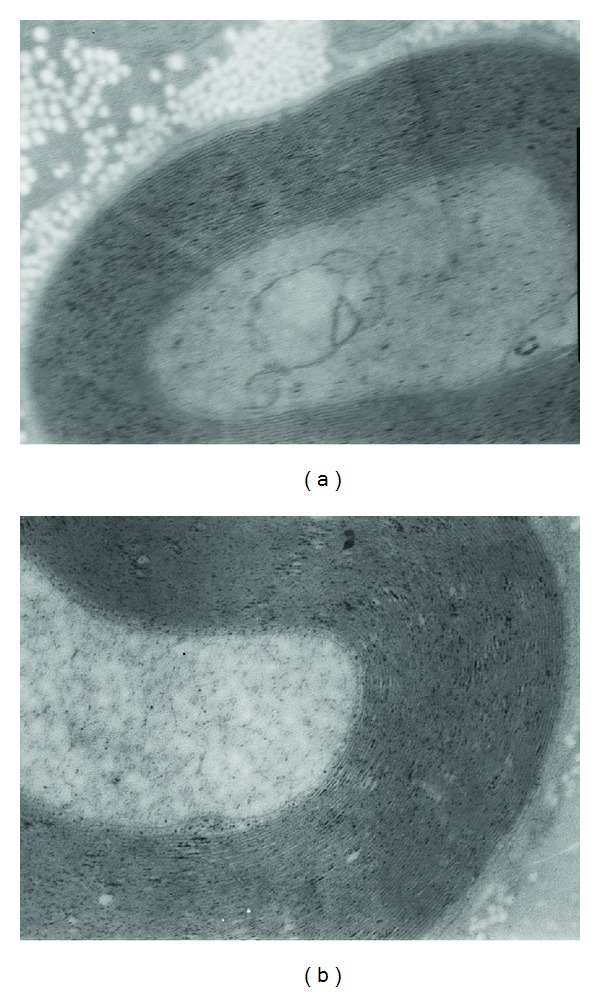
The image of sections stained with uranyl aceate and lead citrate under transmission electron microscope after 16 weeks ECM ×25K ((a) the control group, (b) the experimental group).

The sections were observed under the transmission electron microscope after stained with uranyl acetate and lead citrate. The section stained at week 16 was showed in Figure 2. At 4 weeks, regular dense lamellar structure was detectable in the experimental group, with a majority of confluence, with the presence of obvious hyperplasia, abundant microfilaments and microtubules, and regular dense medullary cord. At 8 weeks, there were thick myelin sheath, regular dense lamellar structure, and abundant mitochondria, microfilaments, and microtubules in the experimental group. At 16 weeks, there were uniform myelin sheath, dense lamellar structure, and abundant regular mitochondria, microfilaments, and microtubules in the experimental group (Figure 2). At 4, 8, and 16 weeks, significant differences in the thickness of myelin sheath of new myelinated nerve fibers and the diameter of axons were detectable between both groups (*P* < 0.05; Tables 4 and 5).

## 4. Discussion

3D scaffold has been widely used in tissue engineering [22–24]. The microstructure of the 3D scaffold itself could mimic the ECM and induce the adhesion, differentiation, and proliferation of cell. After scaffold implantation, the growth of capillaries into the porous construct may be too slow to provide adequate nutrients to the cell within the scaffold, and this inhibits tissue formation in the scaffold core. Achieving successful vascularization remains one of the main problems in tissue engineering [25, 26].

Vascular endothelial cells are monolayer flat cells, an important component of blood capillary, and a selective permeability barrier between blood and vessel wall. Vascularization contains two mechanisms: angiogenesis and vasiformation. Vasiformation refers to enzymatic hydrolysis of vascular basement membrane, pullulation, proliferation, and then formation of new blood capillary. Moreover, vascular endothelial cells can synthesize and secrete vascular endothelial growth factor and basic fibroblast growth factor, which has effective promoting effects on vascular growth [25–27]. Sahota et al. found that at 1 day after the stent implanted with human microvascular endothelial cells was implanted in nude mouse, endothelial cells migrated in the stent. After 5 days, blood capillary-like conduit appeared. At week 1, microvessel appeared. At week 3, new microvessel was well connected to blood vessel of the host. These confirmed that vascular endothelial cells implanted in biomaterials could proliferate and differentiate into new vessels. Moreover, numerous studies also verified the important effect of vascular endothelial cells in vascularization of tissue engineered bone.

After peripheral nerve injury, local microenvironment suitable for nerve regeneration and nutrient substance needed by nerve regeneration are significant for nerve regeneration, and these are strongly associated with regional blood supply. Early vascularization of graft not only provides enough nutrient substance for Schwann cells and contributes to the growth of new axon but also reduces the production of collagen fiber *in vivo* and is convenient for new axon to traverse. In addition, early vascularization of nerve graft gathers macrophages derived from blood, is beneficial to rapidly clean the degenerated products of myelin sheath and axon after nerve injury, and provides a good channel for the growth of new proximal axon towards the distal end.

Recently, with the development of tissue engineering research, the studies concerning tissue engineered nerve for the repair of nerve defect have been paid great attention by more and more scholars [28–32]. Lots of studies addressed effects of chemically extracted acellular nerve-on-nerve regeneration and functional recovery, but revascularization strongly associated with this process has not been paid attention. Successful cell-based tissue engineering requires a rapid and thorough vascularization in order to ensure long-term implant survival and tissue integration [33, 34], while tissue engineered nerve cannot establish blood circulation in an early stage and restricts its further application in clinic [35, 36].

In the present study, Schwann cells of sciatic nerve of fetal rabbit and vascular endothelial cells of thoracic aorta of fetal rabbit were cocultured at a certain proportion in biodegradable fiber-reinforced 3D scaffolds with satisfactory mechanical properties* in vitro* to form prevascularized nerve complexes. Animal experiment results revealed that the recoveries of foot ulcer healing, nerve conduction velocity, nerve fiber number, and ultrastructure of myelin sheath and regenerating nerve were better in the experimental group than those in the control group. This study provides a new idea for finding a method to promote vascularization of tissue engineered nerve. Nevertheless, the growth of the two kinds of cells *in vivo* and the biocompatibility of vascular endothelial cells and the stent deserve further investigation. It remains poorly understood how to culture abundant Schwann cells and vascular endothelial cells and how the cells interact after implantation. New microvascular formation and precise structure in grafts require further studies.

## 5. Conclusion

The present study demonstrates that the fiber-reinforced 3D composites scaffolds cultured with Schwann cells and vascular endothelial cells can induce vascularized sciatic nerve formation. Prevascularized tissue engineered fiber-reinforced 3D scaffolds for the repair of sciatic nerve defects in rabbits can effectively promote the recovery of neurological function. The composites have satisfactory biocompatibility and are promising to be ideal nerve substitutes, which can promote the regeneration of vessel and nerve.

## Figures and Tables

**Table 1:** Comparison of the nerve conduction velocity at different postoperative time in two groups (*n* = 18, $\bar{x}\pm s$, m/s).

Time (weeks)	Experimental group	Control group	*t *	*P *
4	—	—	—	—
8	23.35 ± 1.05	23.11 ± 0.80	2.13	0.04
16	39.52 ± 1.01	38.34 ± 0.15	2.15	0.04

**Table 2:** Comparison of the wet weight of tibialis anterior muscle at different postoperative time in two groups (*n* = 18, $\bar{x}\pm s$, g).

Time (weeks)	Experimental group	Control group	*t *	*P *
4	2.22 ± 0.13	2.20 ± 0.16	0.40	0.67
8	3.38 ± 0.11	3.28 ± 0.07	2.89	0.01
16	6.10 ± 0.22	5.97 ± 0.15	2.14	0.04

**Table 3:** Comparison of the quantity of nerve fibers at different postoperative time in two groups (*n* = 18, $\bar{x}\pm s$).

Time (weeks)	Experimental group	Control group	*t *	*P *
4	922.97 ± 25.06	901.48 ± 12.39	3.26	0.0025
8	1736.44 ± 36.28	1713.51 ± 25.00	2.20	0.03
16	2840.91 ± 20.93	2823.74 ± 14.42	2.86	0.04

**Table 4:** Comparison of the axon diameter of myelinated nerve fibers at different postoperative time in two groups (*n* = 18, $\bar{x}\pm s$, *μ*m).

Time (weeks)	Experimental group	Control group	*t *	*P *
4	5.83 ± 0.16	5.58 ± 0.42	2.27	0.03
8	6.01 ± 0.09	5.87 ± 0.16	3.09	<0.01
16	6.11 ± 0.08	6.00 ± 0.07	4.62	<0.0001

**Table 5:** Comparison of myelin sheath thickness at different postoperative time in two groups (*n* = 18, $\bar{x}\pm s$, *μ*m).

Time (weeks)	Experimental group	Control group	*t *	*P *
4	1.95 ± 0.14	1.70 ± 0.22	4.10	0.0002
8	1.99 ± 0.11	1.85 ± 0.16	3.10	0.0039
16	2.03 ± 0.05	1.90 ± 0.06	7.16	<0.0001
